# Clopidogrel Versus Prasugrel Versus Ticagrelor in Patients with Acute Coronary Syndrome: New Insights from a Large Registry Compared to Randomized Clinical Trials—A Structured Review

**DOI:** 10.3390/jcm15145714

**Published:** 2026-07-21

**Authors:** Julia Umińska, Tomasz Białoszyński, Michał Skrzypek, Mariusz Gąsior, Piotr Adamski, Robert Gajda, Paul A. Gurbel, Klaudyna Grzelakowska, Tomasz Dominiak, Bartosz Hudzik, Kornelia Kędziora-Kornatowska, Udaya Tantry, Giuseppe Specchia, Łukasz Szarpak, Przemysław Trzeciak, Eliano Pio Navarese

**Affiliations:** 1 Collegium Medicum, Nicolaus Copernicus University, 85-067 Bydgoszcz, Poland; 2 Department of Biostatistics, Faculty of Public Health, Medical University of Silesia, 40-055 Katowice, Poland; 3 Bydgoszcz University of Science and Technology, 85-796 Bydgoszcz, Poland; 4 3rd Department of Cardiology, Faculty of Medical Sciences in Zabrze, Medical University of Silesia, 40-055 Katowice, Poland; 5 Silesian Center for Heart Diseases, 41-800 Zabrze, Poland; 6 Moderndical Technologies Center, 87-100 Torun, Poland; 7 Gajda-Med District Hospital, 06-102 Pultusk, Poland; 8 Sinai Center for Thrombosis Research and Drug Development, Sinai Hospital, Baltimore, MD 21215, USA; 9 Department of Cardiovascular Disease Prevention, Faculty of Public Health in Bytom, Medical University of Silesia, 40-055 Katowice, Poland; 10 University of Pavia, 27100 Pavia, Italy; 11 Institute of Medical Science, Collegium Medicum, The John Paul II Catholic University of Lublin, 20-950 Lublin, Poland; 12 Department of Clinical Research and Development, LUXMED Group, 02-678 Warsaw, Poland; 13 Henry JN Taub Department of Emergency Medicine, Baylor College of Medicine, Houston, TX 77030, USA; 14 Department of Life and Health Sciences, Link Campus University, 00165 Rome, Italy

**Keywords:** clopidogrel, prasugrel, ticagrelor, acute coronary syndrome

## Abstract

**Background:** Due to inconsistent evidence regarding oral P2Y12 inhibitors in patients with ACS, we critically assessed the results of the PL-ACS registry in comparison to the results of randomized clinical trials (RCTs). **Methods:** Publications describing 8 RCTs including 40,724 patients with ACS met the inclusion criteria and were compared with the PL-ACS registry report including 15933 patients after propensity score matching. **Results:** The PL-ACS registry findings were directionally consistent with randomized evidence suggesting better ischemic outcomes with prasugrel- or ticagrelor-based DAPT compared with clopidogrel-based DAPT; however, because of the observational design, propensity score matching, regional availability of selected endpoints, and differences in bleeding ascertainment, these findings should be interpreted as complementary rather than confirmatory. On the other hand, the results of the direct comparison of prasugrel and ticagrelor in the PL-ACS registry, while consistent with the results of the meta-analysis, markedly diverge from the results of the ISAR-REACT 5 study with regard to most efficacy and safety endpoints. **Conclusions:** Randomized evidence and complementary PL-ACS registry data support better ischemic outcomes with prasugrel- or ticagrelor-based DAPT compared with clopidogrel-based DAPT in ACS, although differences in bleeding risk and the observational nature of registry data require cautious interpretation. Current evidence does not establish definitive superiority of either prasugrel or ticagrelor, as direct randomized evidence remains limited and not fully concordant with registry findings.

## 1. Introduction

Following the publication of two landmark randomized clinical trials TRITON–TIMI 38 [1] and PLATO [2] showing the superiority of prasugrel and ticagrelor over clopidogrel, European Society of Cardiology (ESC) guidelines recommend a one-year course of dual antiplatelet therapy (DAPT), composed of aspirin and preferably prasugrel or ticagrelor rather than clopidogrel, unless contraindicated, in patients undergoing percutaneous coronary intervention (PCI) for acute coronary syndrome (ACS); for both prasugrel and ticagrelor-class of recommendation I, level of evidence B [3]. A direct comparison of both potent oral P2Y12 inhibitors in the ISAR-REACT 5 trial [4] demonstrated the superiority of prasugrel treatment over ticagrelor in terms of the primary clinical outcome of death, myocardial infarction (MI) or stroke without an increase in bleeding complications. Based on these results the 2023 ESC guidelines for the management of acute coronary syndromes recommended to consider prasugrel in preference to ticagrelor for ACS patients who proceed to PCI (class of recommendation IIa, level of evidence B) [5]. However, the network meta-analysis of 12 randomized controlled trials (52816 patients) by Navarese et al. [6] suggested the superiority of ticagrelor over prasugrel, as the mortality reduction was observed only with ticagrelor as compared to clopidogrel. Both prasugrel and ticagrelor showed a similar reduction in ischemic events and an increase in bleeding in comparison with clopidogrel [6]. The publication of the PL-ACS registry results including head-to-head comparisons of clopidogrel with prasugrel, clopidogrel with ticagrelor, and prasugrel with ticagrelor provides complementary real-world evidence that may help contextualize, but not replace, randomized evidence when interpreting the comparative effectiveness and safety of oral P2Y12 inhibitors [7].

The purpose of this structured review is to critically evaluate the results of the PL-ACS registry in the context of randomized clinical trials (RCTs) comparing oral P2Y12 inhibitors in patients with acute coronary syndrome (ACS), providing a structured comparison rather than a quantitative evidence synthesis.

## 2. Methods

This study was designed as a structured review with qualitative synthesis, aimed at contextualizing randomized evidence against real-world registry data. The review was conducted in accordance with established methodological principles for evidence synthesis and is reported following the Preferred Reporting Items for Systematic Reviews and Meta-Analyses (PRISMA) statement (Table S4). Data, analytic methods, and study materials supporting the findings are available from the corresponding author upon reasonable request.

### 2.1. Review Question and PICO Framework

The review question was framed according to the PICO structure. The population comprised patients with acute coronary syndrome, predominantly managed invasively. The interventions and comparators were oral P2Y12 inhibitor-based dual antiplatelet therapy strategies including clopidogrel, prasugrel, and ticagrelor. The outcomes of interest were all-cause death, myocardial infarction, stroke, composite ischemic outcomes, bleeding, and net clinical outcome when available. The primary objective was to summarize and qualitatively compare randomized evidence on oral P2Y12 inhibitors in ACS. The secondary objective was to contextualize the PL-ACS registry findings against this randomized evidence, without treating registry-derived estimates as equivalent to randomized comparisons.

### 2.2. Search Strategy

A literature search was performed in PubMed, MEDLINE, EMBASE, Cochrane Central Register of Controlled Trials (CENTRAL), and ClinicalTrials.gov from database inception to 22 April 2026. The last search was performed on 22 April 2026 for all sources. The search combined controlled vocabulary and free-text terms related to oral P2Y12 inhibitors and acute coronary syndrome, using Boolean operators. The core search string was: (*acute coronary syndrome* OR *ACS* OR *myocardial infarction* OR *STEMI* OR *NSTEMI* OR *unstable angina)* AND (*clopidogrel* OR *prasugrel* OR *ticagrelor* OR *P2Y12 inhibitor*) AND (*randomized controlled trial* OR *randomized trial* OR *trial*). Searches were limited to studies in humans and English-language publications. No date restriction was applied. ClinicalTrials.gov was searched using the terms-Condition/disease: *acute coronary syndrome* OR *ACS* OR *myocardial infarction* OR *STEMI* OR *NSTEMI* OR *unstable angina*; Intervention/treatment: *clopidogrel* OR *prasugrel* OR *ticagrelor* OR *P2Y12 inhibitor*, to identify completed randomized trials and verify trial registration details. In addition, reference lists of included articles and relevant reviews/meta-analyses were manually screened to identify potentially eligible studies not retrieved through database searches. Records were imported into a reference-management file, duplicates were removed before screening, and the remaining records were screened independently by two investigators.

### 2.3. Eligibility Criteria

Studies were considered eligible if they met all of the following criteria: (1) randomized controlled trials investigating oral P2Y12 inhibitors (clopidogrel, prasugrel, or ticagrelor) in ACS patients; (2) ≥50% of patients undergoing percutaneous coronary intervention (PCI); (3) reporting clinical outcomes; (4) follow-up duration of at least 12 months.

Studies were excluded if they: (1) compared different regimens of the same P2Y12 inhibitor; (2) were pharmacokinetic/pharmacodynamic studies primarily focused on platelet reactivity; (3) used a crossover design; (4) had a non-randomized design.

Prespecified endpoints included efficacy outcomes (all-cause death, myocardial infarction [MI], stroke, and their composite), safety outcome (bleeding), and net clinical outcome (composite of all-cause death, MI, stroke, and bleeding).

Results from randomized trials were compared with findings from the Polish Registry of Acute Coronary Syndromes (PL-ACS) [7], including analyses of the overall population and propensity score-matched (PSM) cohorts. The PL-ACS registry was treated as complementary observational evidence and was not assigned evidentiary weight equivalent to randomized trials. Importantly, different PL-ACS source populations were used for different endpoints: all-cause mortality was assessed in the national cohort, whereas myocardial infarction, stroke, bleeding, and composite outcomes were available only for the Silesia regional subset. Therefore, comparisons between PL-ACS and RCTs were interpreted qualitatively, with explicit consideration of differences in source populations, endpoint ascertainment, bleeding definitions, treatment allocation, residual confounding, and confounding by indication despite propensity score matching.

### 2.4. Study Selection

**Figure 1:**
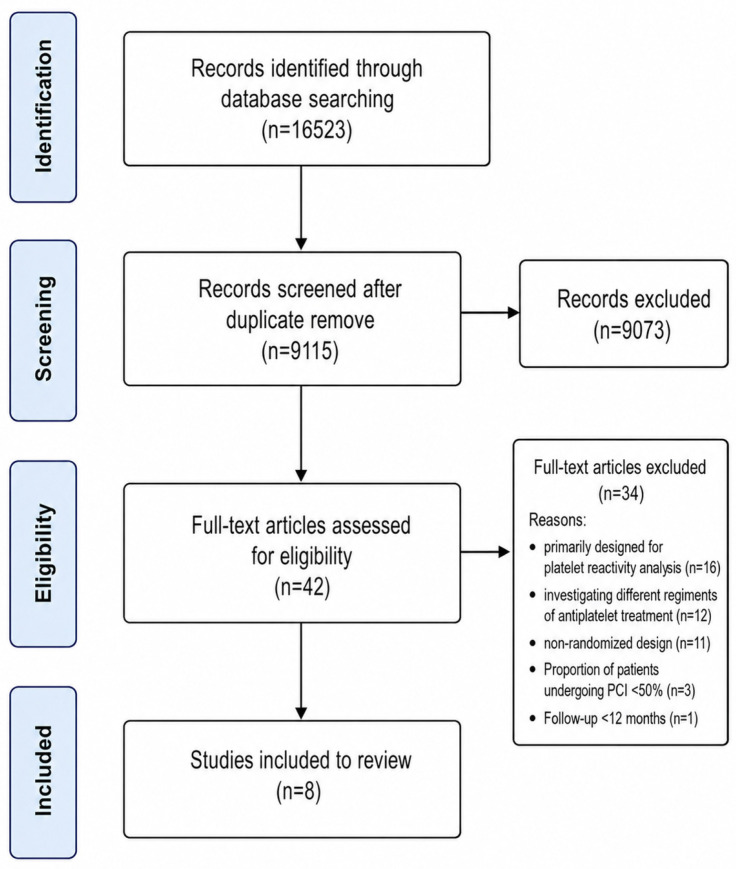
Clopidogrel versus prasugrel versus ticagrelor in patients with acute coronary syndrome. New insights from a large registry compared to randomized clinical trials—a structured review.

Two investigators independently screened titles and abstracts identified through the search strategy. Full-text articles of potentially eligible studies were then assessed independently for inclusion. Duplicate records were removed prior to screening. Only English-language publications were included. Disagreements were resolved by discussion and, when necessary, consultation with a third investigator. The study selection process is presented in the PRISMA flow diagram (Figure 1). When multiple publications, conference reports, interim analyses, or secondary analyses referred to the same randomized trial population, the primary peer-reviewed publication reporting 12-month clinical outcomes was selected as the main source. Conference abstracts and trial registry records were used only to verify trial identity, design, and publication status, and were not treated as independent studies. Duplicate reports and secondary analyses from the same trial population were excluded to avoid double counting.

### 2.5. Data Extraction

Two investigators independently extracted data using prespecified forms. Extracted variables included study design, population characteristics, sample size, treatment arms, follow-up duration, and reported efficacy and safety outcomes. Discrepancies were resolved by consensus with involvement of a third investigator when required.

### 2.6. Quality Assessment

The risk of bias in the included randomized controlled trials was assessed independently by two investigators using the revised Cochrane Risk of Bias tool for randomized trials (RoB 2) (Table S1). The following domains were evaluated: bias arising from the randomization process, bias due to deviations from intended interventions, bias due to missing outcome data, bias in measurement of the outcome, and bias in selection of the reported result. Each trial was classified as having low risk of bias, some concerns, or high risk of bias. Disagreements were resolved by consensus or consultation with a third investigator. Overall, the included trials were of high methodological quality, although several open-label designs introduced a risk of performance bias, and allocation concealment was unclear in some smaller studies.

The PL-ACS registry was assessed separately as an observational comparative study using the ROBINS-I framework (Table S2), with evaluation of confounding, selection of participants, classification of interventions, deviations from intended interventions, missing data, outcome measurement, and selection of reported results. Because registry-based comparisons remain susceptible to residual and unmeasured confounding despite propensity score matching, the PL-ACS evidence was not considered equivalent to randomized evidence.

Detailed results are provided in the Supplementary Material.

### 2.7. Outcomes

The prespecified efficacy endpoints were all-cause death, myocardial infarction, stroke, and the composite of all-cause death, myocardial infarction, or stroke. The safety endpoint was bleeding, as defined in the individual trials.

### 2.8. Synthesis of Evidence

Given substantial clinical and methodological heterogeneity across the included trials—including differences in bleeding definitions (TIMI, PLATO, BARC), study design (double-blind vs. open-label), and patient populations—a pooled quantitative meta-analysis was not performed, as it could introduce bias and yield potentially misleading summary estimates.

Instead, the evidence was synthesized using a structured qualitative approach, with findings from randomized trials contextualized against results from the PL-ACS registry, including propensity score-matched analyses where available. This approach was chosen to place real-world observations within the framework of randomized evidence rather than to generate a new pooled effect estimate.

The synthesis was prespecified around three pairwise comparisons: prasugrel versus clopidogrel, ticagrelor versus clopidogrel, and prasugrel versus ticagrelor. For each comparison, randomized evidence was considered first, followed by a contextual comparison with registry findings. Differences between randomized and observational results were interpreted with respect to risk of bias, confounding, endpoint definitions, treatment exposure, population characteristics, and outcome ascertainment, in line with the applied risk-of-bias and certainty-of-evidence frameworks.

Propensity score matching in the PL-ACS registry was performed in the original registry analysis to reduce measured baseline differences between treatment groups. The propensity score model included clinically relevant baseline characteristics potentially associated with both treatment selection and outcomes, including demographic variables, ACS type, cardiovascular risk factors, comorbidities, previous cardiovascular history, in-hospital management, and PCI-related variables when available. Patients were matched using a nearest-neighbor approach without replacement. Post-matching balance was assessed by comparing baseline characteristics across treatment groups, and the matched cohorts showed substantially improved balance compared with the unmatched population. Nevertheless, propensity score matching can adjust only for measured covariates; therefore, residual confounding, confounding by indication, and unmeasured differences in treatment selection remain possible.

### 2.9. Reporting Bias and Certainty of Evidence

Given the qualitative nature of the synthesis and absence of pooled effect estimates, formal assessment of reporting bias was not performed. The certainty of evidence was assessed using the GRADE framework (Table S3) and is summarized in the Supplementary Material.

### 2.10. Protocol

The review was not prospectively registered. This is acknowledged as a limitation. Given the qualitative design and absence of quantitative pooling, formal assessment of reporting bias was not performed. However, certainty of evidence was assessed using the GRADE framework.

## 3. Results

Of 16,523 records identified through database searching, publications reporting 8 randomized controlled trials (RCTs) comprising 40,724 patients met the inclusion criteria. Of these, 17,716 patients were treated with clopidogrel, 10,166 with prasugrel, and 12,842 with ticagrelor. The main reasons for exclusion at the full-text stage included non-randomized design, pharmacodynamic or platelet-reactivity-focused studies, comparisons of different regimens of the same P2Y12 inhibitor, crossover design, follow-up shorter than 12 months, fewer than 50% of patients undergoing PCI, duplicate or secondary reports from the same trial population, and absence of relevant clinical outcomes. The study selection process, including detailed reasons for full-text exclusion, is presented in the PRISMA flow diagram.

Two trials (15,051 patients) compared prasugrel with clopidogrel, four trials (20,425 patients) compared ticagrelor with clopidogrel, and two trials (5248 patients) compared prasugrel with ticagrelor [1,2,4,8,9,10,11,12].

The PL-ACS registry covered the period from 1 January 2009, to 31 December 2022. The incidence of all-cause death was assessed in the nationwide cohort (PL population, *n* = 381,278), whereas secondary efficacy and safety endpoints (including myocardial infarction, stroke, and bleeding) were available only for the Silesia region (SL population, *n* = 63,248). For the purpose of comparison with randomized trials, propensity score-matched (PSM) populations were used, including 15,933 patients in the nationwide cohort (PL-ACS PSM-PL) and 2949 patients in the regional cohort (PL-ACS PSM-SL), with equal distribution across treatment groups. Baseline characteristics of patients included in RCTs and the PL-ACS registry are presented in Table 1.

### 3.1. Clopidogrel

Patients treated with clopidogrel constituted the largest group among RCTs (*n* = 17,716 across 6 trials). In the propensity score-matched PL-ACS cohorts, 5311 patients received clopidogrel in the nationwide population and 983 in the Silesia cohort.

The incidence of all-cause death in the PL-ACS registry was 5.5% in the nationwide cohort and 5.7% in the Silesia cohort, which is broadly comparable to the results of large RCTs such as TRITON–TIMI 38 and PLATO, although higher mortality rates were observed in selected smaller or higher-risk populations (e.g., Wang et al. [9]). Bleeding rates varied substantially across RCTs (2.7–14.7%), reflecting differences in definitions and study populations, while PL-ACS estimates (8.2% in the Silesia cohort) were within this range. The composite endpoint of death, myocardial infarction, and stroke in the PL-ACS registry (11.2% in the Silesia cohort) was consistent with rates reported in the largest randomized trials (Table 2).

### 3.2. Prasugrel

Prasugrel was administered to 10,166 patients across 4 RCTs. In the PL-ACS PSM cohorts, 5311 patients in the nationwide cohort and 983 in the Silesia cohort received prasugrel.

All-cause mortality in the PL-ACS registry was 4.4% in the nationwide cohort and 4.5% in the Silesia cohort, which is generally comparable to or slightly higher than that observed in most RCTs. The incidence of the composite endpoint (8.3%) and bleeding (4.9%) in the Silesia cohort was within the range reported in randomized studies, although direct comparisons are limited by heterogeneity in endpoint definitions (Table 2).

### 3.3. Ticagrelor

A total of 12,842 patients received ticagrelor across 6 RCTs. In the PL-ACS PSM cohorts, ticagrelor was administered to 5311 patients in the nationwide cohort and 983 in the Silesia cohort.

All-cause mortality in the PL-ACS registry was 3.9% in both cohorts, which is comparable to or lower than that reported in most RCTs. The incidence of the composite endpoint (7.3%) and bleeding (2.3%) in the Silesia cohort was numerically lower than in many randomized studies, although interpretation should take into account differences in bleeding definitions and patient characteristics (Table 2).

### 3.4. Prasugrel Versus Clopidogrel

The **TRITON–TIMI 38 trial** was the first large randomized study in patients with acute coronary syndrome (ACS) undergoing percutaneous coronary intervention (PCI) comparing prasugrel with standard-dose clopidogrel [1]. Prasugrel (60 mg loading dose followed by 10 mg daily) significantly reduced the primary composite endpoint of death from any cause, nonfatal myocardial infarction (MI), or nonfatal stroke by 17% compared with clopidogrel (300 mg loading dose followed by 75 mg daily; HR 0.83, *p* < 0.001). This benefit was primarily driven by a reduction in MI (24%, *p* < 0.001), urgent target-vessel revascularization (34%, *p* < 0.001), and stent thrombosis (52%, *p* < 0.001). The trial was not powered to detect differences in all-cause mortality. Importantly, the reduction in ischemic events with prasugrel was accompanied by a higher risk of bleeding, with a 31% increase in TIMI major or minor bleeding (*p* = 0.002). In predefined high-risk subgroups, including patients aged ≥75 years, those with body weight <60 kg, and those with prior cerebrovascular events, prasugrel was associated with less favorable outcomes [1] (Table 3).

The **Elderly ACS II trial**, which evaluated reduced-dose prasugrel (5 mg) versus standard-dose clopidogrel in elderly patients (>74 years) undergoing PCI, showed no significant differences in the primary composite endpoint or individual efficacy and safety outcomes between treatment groups. The study was terminated prematurely due to futility for efficacy [10] (Table 3).

In the **PL-ACS registry**, propensity score-matched analyses indicated that prasugrel was associated with a lower risk of all-cause mortality and the composite endpoint of death, MI, or stroke compared with clopidogrel. A consistent directional reduction was also observed for MI and bleeding, whereas no significant difference was found for stroke. Detailed estimates are presented in Table 3.

### 3.5. Ticagrelor Versus Clopidogrel

The **PLATO trial** was the largest randomized study comparing two oral P2Y12 inhibitors in patients with acute coronary syndrome (ACS) [2]. This multicenter, double-blind, randomized trial was designed to compare ticagrelor (180 mg loading dose followed by 90 mg twice daily) with clopidogrel (300- to 600 mg loading dose followed by 75 mg daily) for the prevention of cardiovascular events in patients hospitalized with ACS. Ticagrelor significantly reduced the incidence of the primary composite endpoint (death from vascular causes, myocardial infarction [MI], or stroke) by 16% (*p* < 0.001). Furthermore, ticagrelor was associated with a significant reduction in all-cause mortality by 22% (*p* < 0.001), MI by 16% (*p* = 0.005), and stent thrombosis by 33% (*p* = 0.009). Importantly, the improved efficacy of ticagrelor was not accompanied by an increase in overall major bleeding compared with clopidogrel [2] (Table 4).

The **PHILO study**, conducted in an East Asian population (Japan, South Korea, and Taiwan), was designed to assess the consistency of ticagrelor effects with those observed in PLATO [8]. Although not powered to detect statistically significant differences between treatment groups, the study provided important insights into regional variability. Notably, patients enrolled in PHILO had lower body weight compared with those in PLATO, which may have resulted in higher exposure to potent antiplatelet agents and contributed to differences in safety outcomes, including a higher bleeding risk in the ticagrelor group (Table 4) [2,8].

The randomized study by **Wang et al.** evaluated the efficacy and safety of ticagrelor versus clopidogrel in elderly Chinese patients with ACS receiving background aspirin therapy [9]. Despite the limited sample size, ticagrelor was associated with a numerically lower risk of the primary composite endpoint (cardiovascular death, MI, or stroke), as well as reductions in cardiovascular death and MI. However, most comparisons did not reach statistical significance, reflecting limited statistical power. The improved efficacy profile was not associated with a significant increase in bleeding (Table 4) [9].

The **TICAKOREA trial**, a multicenter, investigator-initiated, open-label randomized study, evaluated standard-dose ticagrelor versus clopidogrel in Korean patients with ACS [12]. Ticagrelor was associated with a significantly higher risk of clinically relevant bleeding compared with clopidogrel, while no significant differences were observed in efficacy endpoints (Table 4).

In the **PL-ACS registry**, reflecting real-world clinical practice, propensity score-matched analyses demonstrated that ticagrelor was associated with a lower risk of all-cause mortality and the composite endpoint of death, MI, or stroke compared with clopidogrel. A consistent reduction was also observed for MI and bleeding, whereas no significant difference was found for stroke. Detailed effect estimates are provided in Table 4.

### 3.6. Prasugrel Versus Ticagrelor

The **PRAGUE-18 trial** was the first randomized, multicenter, head-to-head comparison of prasugrel and ticagrelor in patients with acute myocardial infarction undergoing primary percutaneous coronary intervention [11]. The trial was terminated prematurely due to futility, and no significant differences between treatment groups were observed for efficacy or safety endpoints (Table 5).

The **ISAR-REACT 5 trial** was an investigator-initiated, phase 4, multicenter, open-label randomized study comparing ticagrelor and prasugrel in patients with acute coronary syndrome [4]. At 12 months, prasugrel was associated with a significantly lower incidence of the composite endpoint of death, myocardial infarction, or stroke compared with ticagrelor (6.9% vs. 9.3%; *p* = 0.006). This reduction in ischemic events was not accompanied by an increased risk of bleeding (Table 5).

In contrast, findings from the **PL-ACS registry** reflecting real-world practice demonstrated a significantly lower risk of bleeding with ticagrelor compared with prasugrel in propensity score-matched analyses. No significant differences were observed between the two treatments in all-cause mortality or ischemic endpoints, including the composite of death, myocardial infarction, or stroke. Detailed estimates are presented in Table 5.

## 4. Discussion

Given the inherent differences between randomized trials and real-world registries, comparative analyses may provide complementary insights, although they require careful methodological and interpretive caution. We have recently published an analysis of the PL-ACS registry findings, addressing this need [7]. In our view, these results should be related to the existing scientific evidence from randomized clinical trials [1,2,4,8,9,10,11,12] as well as to the previously published meta-analysis of these trials [6].

Differences between registry findings and randomized trials may arise from multiple sources, including residual confounding, confounding by indication, differences in endpoint definitions and adjudication, treatment adherence, and patient selection. Randomized trials remain the gold standard for causal inference, whereas registry data primarily provide insights into effectiveness in routine clinical practice. Therefore, discrepancies between these sources of evidence should not be interpreted as contradictions, but rather as reflections of fundamentally different methodological frameworks.

The PL-ACS registry findings were directionally consistent with two landmark trials, i.e., the TRITON–TIMI 38 [1] and the PLATO [2], showing higher efficacy of DAPT with aspirin and either prasugrel or ticagrelor compared to DAPT with aspirin and clopidogrel [7]. Moreover, for both prasugrel and ticagrelor, the reductions in the efficacy endpoints, both composite endpoint of all-cause death, MI, or stroke and the single endpoint of all-cause death, in the PL-ACS were greater than in both of these randomized clinical trials [1,2] and in the meta-analysis [6]. Other smaller randomized clinical trials were not powered to show significant differences in this regard [8,9,10,12]. The higher efficacy of prasugrel and ticagrelor observed in the PL-ACS registry compared to randomized studies was not accompanied by a significantly higher bleeding tendency [7].

The results of RCTs conducted in the Far Eastern populations [8,9,12] should be acknowledged due to the clear differences, mainly concerning the incidence of bleeding as compared to the PLATO study [2] and the PL-ACS registry [7]. These differences could be attributed to genetic differences that determine the response to oral P2Y12 inhibitors described as the ‘East Asian paradox’ [13,14]. Recognizing these concerns, the World Heart Federation published an expert consensus statement to determine the antiplatelet treatment strategies that are most appropriate for East Asian patients [15].

While the results of the PL-ACS registry are generally consistent with the largest randomized trials and with the results of the meta-analysis regarding the direction of the observed differences between strong oral P2Y12 inhibitors and clopidogrel, the results of the direct comparison of prasugrel with ticagrelor in the ISAR-REACT 5 study markedly diverge from the PL-ACS registry for most of the analyzed endpoints [1,2,4,6,7]. Although the differences observed in ISAR-REACT 5 and the PL-ACS registry did not reach statistical significance for all single efficacy endpoints, the direction of these differences in both studies was opposite in all cases except stroke [4,7]. The ISAR-REACT 5 trial showed the superiority of prasugrel compared to ticagrelor in terms of efficacy assessed by the composite endpoint of all-cause death, myocardial infarction, or stroke, whereas the PL-ACS registry showed the opposite trend. On the other hand, the PL-ACS registry demonstrated the superiority of ticagrelor over prasugrel in reference to safety as assessed by bleeding events, which was consistent with the meta-analysis but not with the results of the ISAR-REACT 5 study [4,6,7]. It is important to note that an RCT like ISAR-REACT 5 follows strict drug administration protocols, while a registry reflects a routine clinical practice, in which the drug administration regimens may vary, potentially impacting the studied outcomes [16,17,18,19,20,21]. The PL-ACS possibly encompasses a more diverse population, including higher-risk patients, not a selected group chosen based on pre-defined criteria, which may also influence both the safety and efficacy analyses. Additionally, the observed divergence between the findings may be attributed to factors such as clinical outcomes adjudication process and geographical differences (the PL-ACS included exclusively Polish patients, while ISAR-REACT 5 was conducted in Germany and Italy).

The overall treatment effect, defined as the net clinical outcome appears to be the optimal comprehensive approach to therapy evaluation. The PL-ACS registry showed associations suggesting more favorable net clinical outcomes of prasugrel and ticagrelor over clopidogrel with respect to net clinical outcome; moreover, ticagrelor was also shown to be superior to prasugrel; however, these findings should be interpreted as observational and hypothesis-generating. [7]. Nevertheless, the net clinical outcome has not been addressed in previous clinical trials, which prevents reference to the results of the PL-ACS registry.

Although the large sample size of the PL-ACS registry increases the precision of estimates and enhances external validity, its observational design means that the findings should be interpreted as complementary to, rather than equivalent to, randomized evidence [7]. The consistency of the PL-ACS registry results with the largest randomized clinical trials (comprising a total of 32,232 patients) and with a previously published meta-analysis (comprising 52,816 patients) further supports the external consistency of these observations [1,2,6,7]. The methodology of conducting registries, including the PL-ACS, is evidently different from randomized clinical trials. It is important to interpret registry data alongside RCTs, as they serve as complementary sources of evidence. Directional consistency between randomized trials and registry findings may increase clinical plausibility, but it should not be interpreted as strengthening causal inference in a straightforward manner, because registry-based estimates remain vulnerable to residual confounding, confounding by indication, differences in endpoint ascertainment, and non-adjudicated or differently defined bleeding outcomes [22,23,24,25]. These discrepancies highlight the need for caution when translating the ISAR-REACT 5 findings into broad clinical preference statements and support the need for further comparative evidence [4,7,26].

This review has several limitations. First, although a structured search and study selection process were applied, the review was not prospectively registered, and reporting bias could not be formally assessed due to the absence of pooled quantitative synthesis. Second, a meta-analysis was not performed because of substantial clinical and methodological heterogeneity across studies, including differences in patient populations, endpoint definitions, and bleeding classifications. In particular, bleeding outcomes were defined using different criteria (TIMI, PLATO, BARC, and patient-reported bleeding in the PL-ACS registry), and composite endpoints were not consistently constructed or adjudicated across trials and registry analyses. As a result, cross-study comparisons should be interpreted as descriptive and contextual rather than as directly comparable estimates of treatment effect, which further justifies the decision not to perform quantitative pooling. Third, several included trials were open-label, and smaller studies were underpowered for clinical endpoints. Finally, comparisons between randomized trials and the PL-ACS registry require careful interpretation, as registry-based estimates remain susceptible to residual confounding despite propensity score matching and cannot be considered equivalent to randomized evidence.

## 5. Conclusions

In patients with acute coronary syndrome, randomized evidence supports the superiority of prasugrel- and ticagrelor-based dual antiplatelet therapy over clopidogrel for ischemic outcomes, although bleeding risk requires individualized assessment. The PL-ACS registry provides complementary real-world observations that are directionally consistent with this evidence, but its observational design, regional availability of selected endpoints, and differences in outcome ascertainment limit causal interpretation.

The comparative effectiveness and safety of prasugrel versus ticagrelor remain uncertain. Direct head-to-head randomized evidence is limited and not fully concordant with registry findings or meta-analytic estimates. Therefore, the discrepancies between ISAR-REACT 5 and PL-ACS should be interpreted cautiously and should not be used alone to draw definitive conclusions regarding the universal preference of one potent P2Y12 inhibitor over the other.

Further adequately powered head-to-head randomized trials and high-quality real-world studies with harmonized endpoint definitions are needed to clarify the relative clinical benefits and safety profiles of prasugrel and ticagrelor.

## Figures and Tables

**Table 1:** Patients’ characteristics in randomized clinical trials and the PL-ACS registry.

STUDY	Publication	Follow-Up	Arm	n	Age	STEMI	NSTEMI	UA	PCI
TRITON-TIMI [NCT00097591]	Wiviott et al., 2007 NEJM [1]	15 m	prasugrel	6813	61	1771 (26%)	5042 (74%)	0	6745 (99%)
			clopidogrel	6795	61	1767 (26%)	5028 (74%)	0	6727 (99%)
PLATO [NCT00391872]	Wallentin et al., 2009 NEJM [2]	12 m	ticagrelor	9333	62	3496 (37.5%)	4005 (42.9%)	1832 (19.6%)	5687 (60.9%)
			clopidogrel	9291	62	3530 (38.0%)	3950 (42.5%)	1811 (19.5%)	5676 (61.1%)
PHILO [NCT01294462]	Goto et al., 2015 Circulation J [8]	12 m	ticagrelor	401	67	205 (51.1%)	66 (16.5%)	129 (32.2%)	340 (84.8%)
			clopidogrel	400	66	210 (52.5%)	74 (18.5%)	116 (29.1%)	338 (84.5%)
Wang et al.	Wang et al., 2016 TCRM [9]	12 m	ticagrelor	100	79	37 (37%)	44 (44%)	19 (19%)	75 (75%)
			clopidogrel	100	80	32 (32%)	47 (47%)	21 (21%)	71 (71%)
Elderly ACS II [NCT01777503]	Savonitto et al., 2018 Circulation [10]	12 m	prasugrel	713	80	298 (42%)	344 (48%)	71 (10%)	707 (99%)
			clopidogrel	730	80	297 (41%)	350 (47%)	83 (12%)	726 (99.5%)
PRAGUE-18 [NCT02808767]	Motovska et al., 2018 JACC [11]	12m	prasugrel	634	61.8	568 (89.6%)	33 (5.2%)	33 (5.2%)	629 (99.2%)
			ticagrelor	596	61.8	533 (89.4%)	34 (5.7%)	29 (4.87%)	591 (99.2%)
TICAKOREA [NCT02094963]	Duk-Woo Park et al., 2019 Circulation [12]	12 m	ticagrelor	400	62.5	170 (42.5%)	148 (37%)	82 (20.5%)	326 (81.5%)
			clopidogrel	400	62.3	156 (39%)	155 (38.8%)	89 (22.2%)	342 (85.5%)
ISAR-REACT 5 [NCT01944800]	Schupke et al., 2019 NEJM [4]	12 m	ticagrelor	2012	64.5	833 (41.4%)	930 (46.2%)	249 (12.4%)	1676 (83.4%)
			prasugrel	2006	64.6	820 (40.9%)	925 (46.1%)	261 (13.0%)	1701 (84.8%)
PL-ACS (PSM-PL)	Umińska et al. [7]	12 m	clopidogrel	5311	62.4	1738 (32.7%)	2550 (48.0%)	1023 (19.3%)	5207 (98.0%)
			prasugrel	5311	62.9	1742 (32.8%)	2539 (47.8%)	1030 (19.4%)	5208 (98.1%)
			ticagrelor	5311	62.9	1729 (32.6%)	2583 (48.6%)	999 (18.8%)	5207 (98.0%)
PL-ACS (PSM-SL)	Umińska et al. [7]	12 m	clopidogrel	983	64.4	300 (30.5%)	432 (43.9%)	251 (25.5%)	945 (96.1%)
			prasugrel	983	64.3	311 (31.6%)	431 (43.8%)	241 (24.5%)	973 (99.0%)
			ticagrelor	983	64.5	291 (29.6%)	446 (45.4%)	246 (25.0%)	973 (99.0%)

TRITON–TIMI 38—Trial to Assess Improvement in Therapeutic Outcomes by Optimizing Platelet Inhibition with Prasugrel–Thrombolysis in Myocardial Infarction 38, PLATO—PLATelet inhibition and patient Outcomes, PHILO—Phase the International Study of Ticagrelor and Clinical Outcomes in Asian ACS Patients, TICAKOREA—Ticagrelor Versus Clopidogrel in Asian/Korean Patients with ACS Intended for Invasive Management, ISAR-REACT 5—Ticagrelor or Prasugrel in Patients with Acute Coronary Syndromes, ACS—acute coronary syndrome, PL-ACS—PL-ACS Registry, PL—population of Poland, SL—population of Silesia region, PSM—Propensity Score Matching, STEMI—ST-elevation myocardial infarction, NSTEMI—non-ST-elevation myocardial infarction, UA—unstable angina, PCI—percutaneous coronary intervention.

**Table 2:** Endpoints in clopidogrel, prasugrel, and ticagrelor arms of randomized clinical trials and the PL-ACS registry.

P2Y12i	Study	Year of Publication	n	Age [Years]	All-Cause Death n (%)	Death, MI, Stroke n (%)	Bleeding n (%)	MI n (%)	Stroke n (%)
Clopidogrel	TRITON-TIMI 38 [1]	2007	6795	61	197 (3.2)	822 (12.7)	231 (3.8) *	620 (9.5)	60 (1.0)
	PLATO [2]	2009	9291	62	506 (5.9)	1065 (12.3)	906 (10.9) **	593 (6.9)	106(1.3)
	PHILO [8]	2015	400	66	7 (1.8)	25 (6.3)	56 (14.7) **	15 (3.8)	6 (1.5)
	Wang et al. [9]	2016	100	79	16 (16)	22 (22)	14(14) **	15 (15)	3 (3.0)
	Elderly ACS II [10]	2018	730	80	28 (3.8)	60 (8.2)	20 (2.7)	19 (2.6)	13 (1.8)
	TICAKOREA [12]	2019	400	62.3	10 (2.5)	27 (6.8)	21(5.3) **	16 (4.0)	5 (1.3)
	PL-ACS (PSM-PL) [7]	2025	5311	62.4	293 (5.5)	na	na	na	na
	PL-ACS (PSM-SL) [7]	2025	983	64.4	56 (5.7)	110 (11.2)	81(8.2) ^#^	59 (6.0)	11 (1.1)
Prasugrel	TRITON-TIMI38 [1]	2007	6813	61	188 (3.0)	692 (10.7)	305(5.0) *	475 (7.3)	61 (1.0)
	Elderly ACS II [10]	2018	713	80	36 (5.0)	57 (8.0)	29(4.1)	14 (2.0)	7 (1.0)
	PRAGUE-18 [11]	2018	634	61.8	30 (4.7)	na	69(10.9) *	19 (3.0)	7 (1.1)
	ISAR- REACT 5 [4]	2019	2006	64.6	73 (3.7)	137 (6.9)	48(4.9) ***	60 (3.0)	19 (1.0)
	PL-ACS (PSM-PL) [7]	2025	5311	62.9	236 (4.4)	na	na	na	na
	PL-ACS (PSM-SL) [7]	2025	983	64.3	44 (4.5)	82 (8.3)	48 (4.9) ^#^	38 (3.9)	7 (0.7)
Ticagrelor	PLATO [2]	2009	9333	62	399 (4.5)	901 (10.2)	946(11.4) **	504 (5.8)	125(1.5)
	PHILO [8]	2015	401	67	10 (2.5)	37 (9.2)	92(23.8) **	24 (6.0)	9(2.2)
	Wang et al. [9]	2016	100	80	9(9)	13(13)	21(21) **	6 (6.0)	2(2.0)
	PRAGUE-18 [11]	2018	596	61.8	25 (4.2)	na	66(11.1) *	15 (2.5)	4 (0.7)
	ISAR-REACT 5 [4]	2019	2012	64.5	90 (4.5)	184 (9.3)	95(5.4) ***	96 (4.8)	22(1.1)
	TICAKOREA [12]	2019	400	62.5	16 (4.1)	37(9.4)	45(11.7) **	20 (5.1)	6(1.6)
	PL-ACS (PSM-PL) [7]	2025	5311	62.9	206 (3.9)	na	na	na	na
	PL-ACS (PSM-SL) [7]	2025	983	64.5	38 (3.9)	72 (7.3)	23 (2.3) ^#^	33 (3.4)	9 (0.9)

* Major or minor bleeding, TIMI criteria; ** Major or minor bleeding, PLATO criteria; *** BARC criteria 2,3,5; ^#^ any patient-reported bleeding, na stands for Not Available.

**Table 3:** Comparison of clinical outcomes in ACS patients treated with prasugrel versus clopidogrel.

Study	Clinical Outcome—Prasugrel vs. Clopidogrel									
	All-Cause Death		All-Cause Death, MI, or Stroke		Bleeding		MI		Stroke	
	HR (95%CI)	*p* Value	HR (95%CI)	*p* Value	HR (95%CI)	*p* Value	HR (95%CI)	*p* Value	HR (95%CI)	*p* Value
TRITON-TIMI38 [1]	0.95 (0.78–1.16)	0.64	0.83 (0.75–0.92)	<0.001	1.31 (1.11–1.56)	0.002	0.76 (0.67–0.85)	<0.001	1.02 (0.71–1.45)	0.93
Elderly ACS II [10]	1.33 * (0.80–2.21)	0.26	0.93 * (0.66–1.32)	0.70	1.52 (0.85–3.16)	0.18	0.75 * (0.37–1.51)	0.42	0.55 (0.22–1.37)	0.2
PL-ACS PSM [7]	0.80 (0.68–0.95)	0.012	0.73 (0.55–0.97)	0.032	0.58 (0.40–0.83)	0.003	0.63 (0.42–0.95)	0.028	0.63 (0.24–1.64)	0.34

* HR estimated on the basis of data provided in the publication.

**Table 4:** Comparison of clinical outcomes in ACS patients treated with ticagrelor versus clopidogrel.

Study	Clinical Outcome—Ticagrelor vs. Clopidogrel									
	All-Cause Death		All-Cause Death, MI, or Stroke		Bleeding		MI		Stroke	
	HR (95%CI)	*p* Value	HR (95%CI)	*p* Value	HR (95%CI)	*p* Value	HR (95%CI)	*p* Value	HR (95%CI)	*p* Value
PLATO [2]	0.78 (0.69–0.89)	<0.001	0.84 (0.77–0.92)	<0.001	1.11 (1.03–1.20)	0.008	0.84 (0.75–0.95)	0.005	1.17 (0.91–1.52)	0.22
PHILO [8]	1.42 (0.54–3.74)	ns	1.51 (0.91–2.50)	ns	1.72 (1.23–2.40)	<0.05	1.63 (0.85–3.11)	ns	1.50 (0.54–4.23)	ns
Wang et al. [9]	0.53 (0.24–1.21)	0.13	0.56 (0.28–1.11)	0.09	1.41 (0.71–2.77)	0.31	0.38 (0.19–0.98)	0.045	0.62 (0.10–3.73)	0.61
TICAKOREA [12]	1.65 (0.75–3.63)	0.22	1.42 (0.86–2.33)	0.17	2.26 (1.34–3.79)	0.002	1.28 (0.66–2.47)	0.46	1.25 (0.38–4.09)	0.72
PL-ACS PSM [7]	0.70 (0.58–0.83)	<0.001	0.64 (0.48–0.86)	0.003	0.27 (0.17–0.43)	<0.001	0.55 (0.36–0.84)	0.006	0.81 (0.33–1.96)	0.64

ns stands for Not Significant.

**Table 5:** Comparison of clinical outcomes in ACS patients treated with prasugrel versus ticagrelor.

Study	Clinical Outcome—Prasugrel vs. Ticagrelor									
	All-Cause Death		All-Cause Death, MI, or Stroke		Bleeding		MI		Stroke	
	HR (95%CI)	*p* Value	HR (95%CI)	*p* Value	HR (95%CI)	*p* Value	HR (95%CI)	*p* Value	HR (95%CI)	*p* Value
PRAGUE-18 [11]	1.13 (0.66–1.92)	0.65	na	na	0.99 (0.70–1.38)	0.93	1.19 (0.61–2.35)	0.61	1.65 (0.48–5.65)	0.42
ISAR-REACT 5 [4]	0.81 * (0.60–1.10)	na	0.74 * (0.59–0.93)	0.006	0.89 * (0.66–1.20)	0.46	0.61 * (0.44–0.85)	na	0.85 * (0.47–1.59)	na
PL-ACS PSM [7]	1.15 (0.95–1.38)	0.15	1.14 (0.83–1.57)	0.408	2.12 (1.29–3.49)	0.003	1.16 (0.73–1.85)	0.54	0.78 (0.29–2.10)	0.62

* HR estimated on the basis of data provided in the publication. The publication shows HR for ticagrelor vs. prasugrel, na stands for Not Available.

## Data Availability

The original contributions presented in this study are included in the article/Supplementary Material. Further inquiries can be directed to the corresponding author.

## Supplementary Materials

The following supporting information can be downloaded at: https://www.mdpi.com/article/10.3390/jcm15145714/s1, Table S1: Risk of bias assessment of included randomized controlled trials using RoB 2; Table S2: Risk of bias assessment of the PL-ACS registry using ROBINS-I; Table S3: GRADE summary of certainty of evidence; Table S4: PRISMA 2020 checklist.
