# Supplementary material for: Clopidogrel Versus Prasugrel Versus Ticagrelor in Patients with Acute Coronary Syndrome: New Insights from a Large Registry Compared to Randomized Clinical Trials—A Structured Review
Source: J Clin Med. 2026 Jul 21;15(14):5714. doi: 10.3390/jcm15145714 (PMC13412429; doi:10.3390/jcm15145714)
Supplement: Supplementary file 1 [file jcm-15-05714-s001.zip › jcm-4242786-supplementary.pdf]

Clopidogrel versus prasugrel versus ticagrelor in patients with acute coronary syndrome. New insights from a large registry compared to randomized clinical trials – a structured review.
Supplementary material

Table S1. Risk of bias assessment of included randomized controlled trials using RoB 2

Study	Randomization process	Deviations from intended interventions	Missing outcome data	Measurement of outcomes	Selection of reported results	Overall risk of bias
TRITON–TIMI 38 (Wiviott et al., 2007)	Low	Low	Low	Low	Low	Low
PLATO (Wallentin et al., 2009)	Low	Low	Low	Low	Low	Low
PHILO (Goto et al., 2015)	Low	Low	Low	Low	Low	Low
Wang et al., 2016	Some concerns	Some concerns	Low	Some concerns	Low	Some concerns
Elderly ACS II (Savonitto et al., 2018)	Low	Some concerns	Low	Low	Low	Some concerns
PRAGUE-18 (Motovska et al., 2018)	Low	Some concerns	Low	Low	Low	Some concerns
TICAKOREA (Park et al., 2019)	Low	Some concerns	Low	Low	Low	Some concerns
ISAR-REACT 5 (Schupke et al., 2019)	Low	Some concerns	Low	Low	Low	Some concerns

Legend: Risk of bias was assessed using the Cochrane Risk of Bias 2 (RoB 2) tool. The following domains were evaluated: bias arising from the randomization process, bias due to deviations from intended interventions, bias due to missing outcome data, bias in measurement of the outcome, and bias in selection of the reported result. Each domain was rated as low risk of bias, some concerns, or high risk of bias, leading to an overall judgment for each study.
Open-label designs (e.g., PRAGUE-18, TICAKOREA, ISAR-REACT 5, Elderly ACS II) were judged as having “some concerns” in the domain of deviations from intended interventions, consistent with prior assessments . Overall, most trials were considered at low risk of bias or with some concerns, without studies at high overall risk.

Table S2. Risk of bias assessment of the PL-ACS registry using ROBINS-I

Study	Confounding	Selection of participants	Classification of interventions	Deviations from intended interventions	Missing data	Measurement of outcomes	Selection of reported results	Overall risk of bias
PL-ACS registry, overall population	Serious	Moderate	Moderate	Moderate	Moderate	Moderate	Moderate	Serious
PL-ACS registry, propensity score–matched cohort	Serious	Moderate	Moderate	Moderate	Moderate	Moderate	Moderate	Serious

Legend: Risk of bias in the PL-ACS registry was assessed using the ROBINS-I framework for non-randomized studies of interventions. Although propensity score matching reduced measured baseline imbalance, residual and unmeasured confounding cannot be excluded because treatment allocation was not randomized. Therefore, the overall risk of bias was judged as serious. The registry evidence should be interpreted as complementary and hypothesis-generating rather than equivalent to randomized evidence.

Table S3. GRADE summary of certainty of evidence

Comparison	Evidence base	Main efficacy outcomes	Bleeding/safety outcomes	Main limitations	Certainty of evidence	Interpretation
Prasugrel vs clopidogrel	RCTs supported by PL-ACS registry	Lower risk of composite ischemic outcomes with prasugrel in TRITON–TIMI 38 and directionally	Increased bleeding in TRITON–TIMI 38; no significant increase in PL-ACS	Heterogeneity in populations and bleeding definitions; observational registry data susceptible to residual confounding	Moderate	Prasugrel is likely more effective than clopidogrel for ischemic outcomes, but bleeding risk requires

Comparison	Evidence base	Main efficacy outcomes	Bleeding/safety outcomes	Main limitations	Certainty of evidence	Interpretation
		consistent PL-ACS findings				individualized assessment
Ticagrelor vs clopidogrel	RCTs supported by PL-ACS registry	Lower risk of ischemic outcomes and mortality in PLATO, directionally supported by PL-ACS	Bleeding findings heterogeneous across PLATO, Asian trials, and PL-ACS	Differences in population characteristics, ethnicity, bleeding definitions, and trial size	Moderate	Ticagrelor is likely more effective than clopidogrel for ischemic outcomes, with uncertainty regarding bleeding magnitude
Prasugrel vs ticagrelor	Head-to-head RCTs and PL-ACS registry	ISAR-REACT 5 favored prasugrel for composite ischemic outcome; PRAGUE-18 was neutral; PL-ACS showed no clear efficacy superiority	PL-ACS favored ticagrelor for bleeding; ISAR-REACT 5 showed no significant bleeding difference	Limited number of head-to-head trials; open-label design; heterogeneous results; observational confounding in registry data	Low	Current evidence does not establish definitive superiority of either prasugrel or ticagrelor
PL-ACS registry comparisons	Observational registry with propensity score matching	Directionally supports superiority of potent P2Y12 inhibitors over clopidogrel	Bleeding estimates differ from several RCTs	Non-randomized treatment allocation; residual confounding; differences in outcome ascertainment and endpoint definitions	Low	Registry findings are clinically informative but should be interpreted as complementary to randomized evidence

Legend: Certainty of evidence was assessed using the GRADE framework. Randomized evidence was initially rated as high certainty and downgraded for risk of bias, inconsistency, indirectness, imprecision, or heterogeneity when applicable. Observational evidence from the PL-ACS registry was initially rated as low certainty and was not upgraded because of residual confounding and non-randomized treatment allocation. Certainty ratings were used to guide the strength of interpretation rather than to support causal equivalence between registry and randomized evidence.

Table S4. PRISMA 2020 checklist

Section/Topic	Item	Checklist item	Reported (Yes/No)	Location in manuscript
TITLE	1	Identify the report as a systematic review	Yes	Title
ABSTRACT	2	Structured summary	Yes	Abstract
INTRODUCTION	3	Rationale	Yes	Introduction
INTRODUCTION	4	Objectives	Yes	Introduction
METHODS	5	Eligibility criteria	Yes	Methods
METHODS	6	Information sources	Yes	Methods
METHODS	7	Search strategy	Yes	Methods
METHODS	8	Selection process	Yes	Methods
METHODS	9	Data collection process	Yes	Methods
METHODS	10	Data items	Yes	Methods
METHODS	11	Risk of bias assessment	Yes	Methods; Suppl. Tables S1, S2
METHODS	12	Effect measures	Yes	Methods
METHODS	13	Synthesis methods	Yes	Methods
METHODS	14	Reporting bias assessment	No	Not performed (see Limitations)
METHODS	15	Certainty assessment	Yes	Suppl. Table S3
RESULTS	16	Study selection	Yes	Results; PRISMA flow diagram
RESULTS	17	Study characteristics	Yes	Table 1
RESULTS	18	Risk of bias in studies	Yes	Suppl. Tables S1, S2
RESULTS	19	Results of individual studies	Yes	Tables 2–5
RESULTS	20	Results of syntheses	Yes	Results

Section/Topic	Item	Checklist item	Reported (Yes/No)	Location in manuscript
RESULTS	21	Reporting bias	No	Not performed
RESULTS	22	Certainty of evidence	Yes	Suppl. Table S3
DISCUSSION	23	Summary of evidence	Yes	Discussion
DISCUSSION	24	Limitations	Yes	Discussion
DISCUSSION	25	Interpretation	Yes	Discussion
OTHER	26	Registration and protocol	No	Methods (acknowledged)
OTHER	27	Support	Yes	Manuscript
OTHER	28	Competing interests	Yes	Manuscript
OTHER	29	Availability of data	Yes	Methods
